# Supplementary material for: Impact of Body Mass Index in the Cardioverter Efficacy of Amiodarone in Persistent Atrial Fibrillation
Source: Pharmaceuticals (Basel). 2024 May 28;17(6):693. doi: 10.3390/ph17060693 (PMC11206555; doi:10.3390/ph17060693)
Supplement: Supplementary file 1 [file pharmaceuticals-17-00693-s001.zip › pharmaceuticals-2871518-supplementary.pdf]

Supplementary Table S1. Baseline characteristics of the 467 patients without structural heart disease.

	N (%)	No PCV [illegible]	PCV (n=111)	p
Age (years)	64.02±10.32	64.27±10.37	63.21±10.18	0.345
Male gender	325 (69.6)	225 (70.8)	73 (65.8)	0.315
Diabetes mellitus	70 (15.0)	56 (15.7)	14 (12.6)	0.422
Hypertension	261 (55.9)	207 (58.1)	54 (48.6)	0.078
COPD	47 (10.1)	36 (10.1)	11 (9.9)	0.951
LVEF < 40%	5 (1.2)	4 (1.1)	1 (0.9)	0.834
NYHA ≤ 2	151 (32.4)	117 (33.1)	34 (30.4)	0.660
AF duration > 1	61 (13.1)	52 (14.6)	9 (8.1)	0.076
LA size > 50 mm	50 (11,5)	40 (12.2)	10 (9.0)	0.433
LA size	43.02±5.44	43.50±5.29	41.52±5.77	0.001
LVEF	61.76±7.77	61.41±7.65	62.84±7.95	0.106
BMI	29.09±4.34	29.53±4.38	27.61±3.83	<0.001

AF, atrial fibrillation; BMI, body mass index; COPD, chronic obstructive pulmonary disease; LA, left atrium; LVEF, left ventricular ejection fraction; NYHA, New York Heart Association; PCV, pharmacological cardioversion.

Supplementary Table S2. Parameters associated with reversion to SR before ECV in patients without structural heart disease treated with amiodarone. Multivariate analysis

	OR (95 % CI)	P
BMI	0.899 (0.844-0.959)	0.001
AF duration > 1 year	0.375 (0.142-0.990)	0.048
LA size	0.943 (0.902-0.986)	0.010
Hypertension		0.521

AF, atrial fibrillation; BMI, body mass index; ECV, electrical cardioversion; SR, sinus rhythm.
